# Increased Sensory Processing Atypicalities in Parents of Multiplex ASD Families Versus Typically Developing and Simplex ASD Families

**DOI:** 10.1007/s10803-016-2888-0

**Published:** 2016-08-18

**Authors:** Chelsea K. Donaldson, Johannes E. A. Stauder, Franc C. L. Donkers

**Affiliations:** 1 Department of Cognitive Neuroscience, Faculty of Psychology and Neuroscience, Maastricht University, Maastricht, The Netherlands; 2 Department of Psychiatry, University of North Carolina at Chapel Hill, Chapel Hill, NC USA

**Keywords:** Autism spectrum disorder, Sensory processing, Simplex, Multiplex, Hypersensitivity, Hyposensitivity, Broader autism phenotype

## Abstract

Recent studies have suggested that sensory processing atypicalities may share genetic influences with autism spectrum disorder (ASD). To further investigate this, the adolescent/adult sensory profile (AASP) questionnaire was distributed to 85 parents of typically developing children (P-TD), 121 parents from simplex ASD families (SPX), and 54 parents from multiplex ASD families (MPX). After controlling for gender and presence of mental disorders, results showed that MPX parents significantly differed from P-TD parents in all four subscales of the AASP. Differences between SPX and MPX parents reached significance in the Sensory Sensitivity subscale and also in subsequent modality-specific analyses in the auditory and visual domains. Our finding that parents with high genetic liability for ASD (i.e., MPX) had more sensory processing atypicalities than parents with low (i.e., SPX) or no (i.e., P-TD) ASD genetic liability suggests that sensory processing atypicalities may contribute to the genetic susceptibility for ASD.

## Introduction

Autism spectrum disorder (ASD) is primarily characterized by social/communication deficits and restricted repetitive behaviors (American Psychiatric Association [APA] 2013). Beginning with the first observations of autism (Asperger 1944; Kanner 1943), it has become well known that many individuals with ASD also have abnormal reactions to sensory input, which include hyperresponsiveness, hyporesponsiveness, and sensory seeking behaviors (e.g., Baranek et al. 2006; Tomchek and Dunn 2007). Evidence of sensory processing abnormalities in individuals with ASD has been demonstrated throughout a variety of measurements and samples consisting of children (Adamson et al. 2006; Baranek et al. 2006; Kirby et al. 2015; Leekam et al. 2007; Tomchek and Dunn 2007; Tomchek et al. 2014) and adults (Cascio et al. 2008; Crane et al. 2009; Grandin 1992; Leekam et al. 2007; Tavassoli et al. 2014) with ASD, including physiological evidence showing hyperresponsive brain activity in reaction to sensory stimuli in ASD youth (Green et al. 2013). Consistent with these findings, “hyper- or hypo-reactivity to sensory input or unusual interests in sensory aspects of the environment” was added as one of the four symptom subcategories defining “repeated, repetitive behaviors” of ASD in the newest version of the *Diagnostic and Statistical Manual of Mental Disorders,* 5th *Edition* (DSM-5; APA 2013), thus proposing abnormal sensory processing as a significant feature of ASD.

### Genetic Influences of Abnormal Sensory Processing and ASD

Considering that ASD has moderate to strong genetic influences (Hallmayer et al. 2011; Ritvo et al. 1985), and that sensory processing abnormalities are shown to be moderately heritable (Goldsmith et al. 2006) and highly prevalent in individuals with ASD (Baranek et al. 2006), it has been suggested that sensory processing abnormalities may share genetic influences with the defining characteristics of ASD (DeLorey et al. 2011; Peñagarikano et al. 2011; Tavassoli et al. 2012). Rodent models of ASD have found that several genetic and epigenetic insults known to produce ASD-like symptoms of social impairments and repetitive/stereotypic behaviors in rodents have also resulted in sensory processing abnormalities (for a review, see Argyropoulos et al. 2013). For instance, Peñagarikano et al. (2011) found that a knockout of CNTNAP2, a well-known ASD candidate gene, resulted in increased thermal and olfactory hypersensitivity in the affected mice. Similarly, DeLorey et al. (2011) found that heterozygosity for the ASD candidate gene GABRB3 in rodents was significantly associated with increased thermal and tactile hypersensitivity. Following these studies, Tavassoli et al. (2012) investigated whether hypersensitivity was also related to GABRB3 variations in humans. They found that behavioral and parent-report measurements of tactile hyperresponsivity in typically developing children were associated with common variations in the GABRB3 candidate gene, thus further supporting genetic implications of sensory processing abnormalities in ASD.

In addition to molecular genetics methods, an alternative method of examining whether particular symptoms are related to genetic influences of ASD is through the assessment of these symptoms in parents and relatives of ASD probands. Relatives of individuals with ASD have an increased chance of displaying mild autistic traits (Bailey et al. 1998; Piven et al. 1997; Szatmari et al. 2008; Taylor et al. 2013), described as the “broader autism phenotype” (BAP; Bolton et al. 1994). BAP traits may represent mild phenotypic expressions of the same genetic influences responsible for ASD (Bailey et al. 1998; Piven 2001). Thus, investigating ASD-like traits in relatives can aid in the search for intermediate phenotypes that may contribute to the genetic liability for ASD (Piven 2001).

The likelihood of BAP traits in relatives is higher in families in which multiple members are diagnosed with ASD (multiple-incidence/multiplex families; MPX) compared to families in which only one relative has ASD (single-incidence/simplex families; SPX), which is likely due to differing genetic mechanisms in these families. While some cases of ASD are heavily influenced by spontaneous de novo mutations (rare genetic mutations present in the child but absent in the parents) of large effect producing sporadic cases of ASD (i.e., SPX) (O’Roak et al. 2012; Sebat et al. 2007), others might be due to the inheritance of ASD-related genes producing familial cases of ASD (i.e., MPX) (Virkud et al. 2009). Supporting the former hypothesis, Sebat et al. (2007) found that the rate of de novo copy number variants was significantly higher in ASD probands from SPX (10 %) compared to those from MPX (3 %) families and control families with no diagnoses of autism (1 %).

Additional evidence supporting the genetic distinction between MPX and SPX families derives from several studies showing that ASD-related traits were more common in relatives from MPX families than those from SPX families (e.g., Bernier et al. 2012; Losh et al. 2008), implying that ASD in the former group is strongly influenced by inheritance of familial ASD traits. Szatmari et al. (2000) evaluated ASD traits in 1362 biological relatives of 78 ASD probands and found that social impairments were significantly more prominent in MPX than in SPX relatives (*p* < .001), and MPX relatives were also more likely than SPX to have impairments in two or more areas of the three primary symptom categories of ASD (*p* = .05). Subsequent studies found similar results, showing that, compared to SPX relatives, MPX relatives (usually parents and/or unaffected siblings) had worse pragmatic language and lower quality friendships (Losh et al. 2008), less social interest and less non-verbal communication expression (Gerdts et al. 2013), less social motivation (Bernier et al. 2012), and scored higher on the Social Responsiveness Scale (SRS), signifying more ASD-related deficits (Constantino et al. 2010). A recent study by Oerlemans et al. (2015) also found significant differences in the number of autistic traits between SPX and MPX unaffected siblings, but not between unaffected SPX/MPX parents. Only one study failed to find differences in autistic traits between MPX and SPX families, although they did find marginally significant differences between MPX fathers and control fathers on the SRS (De la Marche et al. 2011). To date, no study has evaluated sensory processing atypicalities in MPX versus SPX families, and therefore it is currently unclear if this common ASD symptom may contribute to the genetic liability for familial cases of ASD.

### Abnormal Sensory Processing in Relatives of Individuals with ASD

Two recent studies have evaluated sensory processing in relatives of ASD individuals (De la Marche et al. 2012; Uljarević et al. 2014), although neither used the MPX/SPX distinction outlined above. Both studies used the adolescent/adult sensory profile (AASP) self-report questionnaire, which consists of four quadrants (subscales) corresponding to different types of sensory reactions: hyposensitivity, hypersensitivity, sensation seeking and sensation avoidance (Brown and Dunn 2002). De la Marche et al. (2012) compared 56 non-affected adolescent siblings of ASD individuals with 33 adolescent control participants, and found similar AASP scores between groups except for the Sensation Seeking quadrant, in which siblings of ASD individuals scored significantly lower than adolescent controls (*p* < .001). The authors concluded that decreased sensation seeking behaviors might be an endophenotypic trait of ASD.

Only one study to our knowledge has investigated sensory processing atypicalities in parents of children with ASD. Uljarević et al. (2014) found that 49 of the 50 mothers (98 %) of children with ASD scored one or more standard deviations (SD) outside the AASP normative means on at least one quadrant of the AASP, and 22 (44 %) scored two or more SDs outside the norms. While Uljarević et al.’s study produced valuable initial discoveries, they only included descriptive findings that compared their sample to the normative means and abnormal classifications found in the AASP manual. As such, a carefully controlled comparison group is needed in order to clearly understand the extent to which sensory processing in parents of ASD children differs from parents of typically developing (TD) children.

In addition, Uljarević et al. (2014) did not control for factors previously associated with scores on self-report sensory processing questionnaires, such as gender and presence of mental disorders, and thus it is unclear if these factors could partially explain their results. Females are known to have a higher sensitivity than males in several modalities (for a review, see Velle 1987). Three recent studies using self-report questionnaires have further supported gender differences in sensory processing, finding that females reported significantly more sensory processing atypicalities than males (Engel-Yeger 2012; Horder et al. 2014; Tavassoli et al. 2014).

Sensory processing abnormalities have been associated with various mental disorders^1^ and disorder symptoms, including ADHD (Mangeot et al. 2001; Ashburner et al. 2008), bipolar disorder (Brown et al. 2002), obsessive–compulsive disorder (OCD; Dar et al. 2012; Rieke and Anderson 2009), post-traumatic stress disorder (PTSD; Engel-Yeger et al. 2013), schizophrenia (Brown et al. 2002), anxiety (Engel-Yeger and Dunn 2011; Horder et al. 2014; Liss et al. 2005) and depression (Liss et al. 2005). In addition, relatives of ASD probands are known to have increased rates of several mental disorders that are associated with sensory symptoms, including OCD (Bolton et al. 1998), affective disorders (Bolton et al. 1998; Piven and Palmer 1999), bipolar disorders (DeLong and Nohna 1994), and schizophrenia (Daniels et al. 2008). Therefore, in order to disentangle influences specific to ASD, it is crucial to control for presence of mental disorders when investigating sensory processing in relatives of individuals with ASD.

### Purpose of the Study

The purpose of the present study was to examine how sensory processing differs in parents of children with ASD from SPX and MPX families compared to parents of TD children, in order to gain further insight into the heritable links between sensory symptoms and ASD. This study sought to expand Uljarević et al.’s (2014) initial findings by comparing parents of ASD children with a control group, including fathers in both groups, and examining confounding variables such as gender and presence of mental disorders. In addition, this study aimed to further differentiate the effects of genetic liability for ASD by separating the parents of ASD children into pre-defined SPX and MPX parent groups.

In the present study, the AASP was used to measure sensory processing in parents to allow for direct comparison of this study’s results with the two past studies exploring atypical sensory processing in relatives of individuals with ASD (De la Marche et al. 2012; Uljarević et al. 2014). Based on past research demonstrating that sensory processing abnormalities are heritable (Goldsmith et al. 2006) and related to ASD candidate genes (DeLorey et al. 2011; Peñagarikano et al. 2011; Tavassoli et al. 2012), it was hypothesized that parents of ASD children who presumably have the highest genetic liability for ASD (MPX) would differ from parents with lower ASD genetic liability (SPX) and parents with no such liability (parents of typically developing children; P**-**TD) in sensory processing scores on all four quadrants of the AASP.

## Methods

### Participants

Three groups of participants were recruited: parents from multiplex ASD families, parents from simplex ASD families, and parents who had a TD child with no biological ASD relatives. Participants were excluded from the study if they had their own diagnosis of ASD (*n* = 8), and one participant was excluded due to extreme outlier scores on two of the outcome measures (through use of the outlier labeling rule of Hoaglin and Iglewicz 1987), which suggested rushing or response bias.

Participants were included in the P-TD group if they were the biological parent of at least one typically developing child 4+ years old. Participants were excluded from the P-TD group if any of their biological children had a developmental disorder (*n* = 16) or if there were suspicions that their child might have ASD (*n* = 12). As this study focused on traits in ASD-affected families compared to TD families, participants in the P-TD group who had a biologically-related first-, second-, or third-degree family member with ASD were also excluded (*n* = 7), thus leaving 85 participants in the P-TD group.

Participants were included in the ASD-affected parent groups (MPX or SPX) if they reported they were the biological parent of at least one child 4+ years old who was diagnosed with ASD by a licensed professional (or a previous DSM classification name, such as Asperger’s syndrome, autism disorder, or pervasive developmental disorder-not otherwise specified). In order to include a wide range of severity, it was not made prerequisite that the ASD child was free of comorbid disorders or intellectual disability.

Participants from the ASD-affected group were designated to the SPX group if they (1) had only one biological child with ASD, (2) had at least one biological child without ASD, and (3) had no biological first-, second- or third-degree relatives with ASD. Therefore, singleton ASD families were excluded from this group (*n* = 48), which left 121 parents in the SPX group. While previous ASD studies included only families with two or more biological ASD siblings in their MPX group (e.g., Losh et al. 2008), we used a more liberal definition of MPX and, in addition to previous criteria, we also included parents who had one biological child with ASD and additionally had one or more first-, second-, or third-degree family members diagnosed with ASD who was biologically related to both the participant and to his or her child with ASD. Although the present study is the first to our knowledge to apply this expanded MPX definition to ASD families, various studies in other fields have used a similar definition of MPX, in which MPX families consisted of two or more first- or second-degree related family members (e.g., Blouin et al. 1998; Michel et al. 2001). As the purpose of the SPX/MPX separation is to represent the underlying genetic distinction between sporadic and familial cases of ASD, we chose this broader definition of MPX because the chances are extremely low that two or more cases of ASD in one biological family are both due to independently occurring rare de novo mutations that similarly resulted in ASD expression. Therefore, by broadening the definition to include any biological relative with ASD, not just a sibling, we maximize the chances of including all familial cases of ASD on the presumption that two cases in one biological family are most likely due to commonly inherited ASD traits. Nevertheless, in order to ensure that this method of distinction was valid, preliminary analyses were run to investigate whether those in the traditional definition of MPX (two or more ASD siblings in one nuclear family; *n* = 30) differed from those in the broader definition (one ASD child + one ASD family member biologically related to the ASD child and the parent; *n* = 24). No significant differences were found between these two groups on any outcome measures (.45 ≤ *p* ≤ .79), and therefore participants were confidently pooled together to form one MPX parent group (*n* = 54).

Table 1 shows the demographics of the P-TD (*n* = 85), SPX (*n* = 121), and MPX (*n* = 54) parent groups. Chi square tests of independence for each demographic variable showed that the three groups did not significantly differ in regard to gender, country of residence, education level, or amount of parents in each group currently diagnosed with a mental disorder (all *p*s > .05). The three groups differed in the amount of parents in the youngest age range (*p* < .05), with the P-TD group reporting more than expected in this youngest range and the MPX group reporting less than expected. However, as age was found to be unrelated to sensory processing scores in young- and middle-aged adult populations in three past studies all using different sensory questionnaires including the AASP (Crane et al. 2009; Robertson and Simmons 2013; Tavassoli et al. 2013), this significant difference in age between the P-TD and MPX groups was not problematic for the present study.

**Table 1:** Participant demographics

Participant characteristics	*χ* ^2^	P-TD (*n* = 85) (%)	SPX (*n* = 121) (%)	MPX (*n* = 54) (%)
Gender				
Female	2.931	81.2	87.6	90.7
Male		18.8	12.4	9.3
Country of residence	2.339			
US		87.4	92.8	93.1
Other		12.6	7.2	6.9
Age group				
21–30 years old	16.128*	18.8*	8.3	1.9*
31–40 years old		40.0	41.3	37.0
41–50 years old		29.4	43.8	50.0
51–61 years old		11.8	6.6	11.1
Highest level of education	2.355			
High school or G.E.D.		5.9	5.8	7.4
Some college/voc. school^a^		30.6	32.2	25.9
Bachelor’s degree		34.1	39.7	37.0
Graduate degree		29.4	22.3	29.6
Presence of mental disorder	5.005			
No		65.9	71.1	53.7
Yes		34.1	28.9	46.3
Type of disorder^b^				
ADHD/ADD		5.9	4.1	7.4
Anxiety disorders		10.6	9.1	18.5
Avoidant personality		1.2	0	0
Bipolar disorders		2.4	3.3	14.8
Depression disorders		21.2	19.8	31.5
OCD		2.4	3.3	3.7
PTSD		1.2	2.5	0.0
SPD		0.0	.8	0.0

*P-TD* parents of typically developing children, *SPX* parents of children with autism spectrum disorder (ASD) from simplex families, *MPX* parents of children with ASD from multiplex families, *ADHD/ADD* attention deficit hyperactive disorder/attention deficit disorder, *OCD* obsessive–compulsive disorder, *PTSD* post traumatic stress disorder, *SPD* sensory processing disorder

^a^Due to the small number of responses in the “vocational school” category, this group was combined with the “some college” category for analyses in order to meet assumptions concerning minimum expected cell count for Chi square tests

^b^The sum percentages of each type of disorder are greater than the total percentage of “presence of mental disorder” due to comorbidities (presence of two or more disorders) in participants

* *p* < .05

Participants were recruited through various methods of asking third party administrators to share the study’s website with potential participants. Recruitment for the majority of the two ASD-affected groups consisted of calling and/or emailing the head of 419 ASD centers, societies, parent support groups, and schools across the United States to request advertisement of the study’s website. Fifty-five groups agreed to participate through either: hanging the study’s flyer in their center; posting the study on their website, social media pages, or online discussion groups; or sending a mass email to all families involved with the organization. The study was also posted on several websites geared toward ASD research or increasing ASD awareness.

Recruitment aiming to gain participants in the P-TD group consisted of convenience sampling by the authors and contacting the head of daycares, kindergarten-12th grade schools, and universities in the United States to advertise the study in the same ways described above. Eight organizations agreed to do so, including two universities who sent mass emails to all their employees. In order to increase sample size, the study was also posted to six websites that aim to recruit research participants. All participants were asked to explain how they found the survey. From this data, it was found that participants from the five recruitment methods (social media post by an organization, flyer, post on a participant recruitment website, convenience sampling, or direct email) did not differ in any outcome measures (all *p*s > .05).

## Materials

### Adolescent/Adult Sensory Profile (AASP)

The AASP is one of the most widely used self-report questionnaires to evaluate sensory processing in adults (Brown and Dunn 2002). This questionnaire is based on Dunn’s (1997) model of sensory processing, which explains reactions to sensory input through a four-quadrant model. One dimension of this model is dedicated to neurological threshold (high threshold: hyposensitive; low threshold: hypersensitive), and the other to behavioral response/self-regulation (active or passive response to the given threshold). There is one subscale for each of the four quadrants, which consist of low registration (LR; high threshold/passive behavioral response), Sensation Seeking (S. Seeking; high threshold/active behavioral response), Sensory Sensitivity (S. Sensitivity; low threshold/passive behavioral response), and Sensation Avoidance (S. Avoidance; low threshold/active behavioral response).

The AASP has 60 items (15 items for each quadrant subscale), which are dispersed throughout six sensory modalities (Taste/Smell, Auditory, Visual, Tactile, Movement, and Activity). Participants respond on an increasing five-point scale how often they behave in the way described by the item (Almost Never, Seldom, Occasionally, Frequently, or Almost Always). Scores for each quadrant range from 15 to 75. An example item is: “I stay away from noisy settings.”

Standard errors of measurement of the AASP range from 3.58 to 4.51 (Brown and Dunn 2002), and internal consistency was found to be good for LR (α = .78), adequate for S. Seeking (α = .60), and good for S. Sensitivity (α = .78) and S. Avoidance (α = .77) (Brown et al. 2001). In the present sample, internal consistencies of the AASP quadrants were similar to Brown et al.’s (2001) results, with good reliability for LR (α = .78), S. Seeking (α = .70), S. Sensitivity (α = .82), and S. Avoidance (α = .84). Supporting concurrent validity of the AASP, scores in the low threshold quadrants (S. Sensitivity and S. Avoidance) were shown to have strong correlations with another self-report questionnaire of hypersensitivity/over-responsivity in adults, the Sensory Over-Responsivity (SensOR) Inventory: SensOR versus S. Sensitivity: *r* = .74; SensOR versus S. Avoidance: *r* = .64; both *p*s < .001 (Schoen et al. 2008).

Using a sample of 496 participants without disabilities aged 18–64 years old, Brown and Dunn (2002) have produced normative means with five classification groups of scores corresponding to how much an individual score differs from the normative mean for each quadrant. The five classification groups follow a normal distribution for each quadrant, for which a score below the 2nd percentile is considered “Much Less than Most People,” a score between the 2nd and 16th percentile is “Less than Most People,” between the 16th and 84th percentile is “Similar to Most People,” between the 84th and 98th percentile is “More than Most People,” and a score above the 98^th^ percentile is “Much More than Most People.”

### Demographics/Background Questionnaire

The demographics questionnaire inquired about general characteristics of the participant, such as gender, age group, and education level, but also characteristics specific to this study, such as number of biological children and family history of ASD. As it was not possible to conduct clinical diagnostic methods due to the online format of this study, participants were asked to personally report if they had ever been diagnosed with any mental disorder, and if so, which disorder(s). They were also asked which disorders, if any, they were currently diagnosed with.

## Procedure

This study was part of a larger project consisting of six questionnaires total, one of which was the AASP. All validated questionnaires, including the AASP, were entered in their original versions into the online software program Qualtrics.com (Qualtrics, LLC 2015). The total survey took approximately 35–40 min, while the parts relevant to the present study, the AASP and demographics questionnaire, took approximately 10–12 min. To be included in the present study, only completion of the AASP and demographics questionnaires was required. The survey link was posted on a one-page website, which included a short description of the study, contact information, and inclusion criteria. Participants who completed the questionnaire were entered in a raffle to win one of two $50 gift cards (or the equivalent amount in the participant’s home currency).

This study was approved by the Ethical Committee Psychology at Maastricht University (ECP-147_10_12_2014). Informed consent was obtained from all individual participants included in the study.

## Data Analyses

Only participants who completed at least 95 % of the required questionnaires were included in data analyses (*n* = 260).

### Descriptive Findings and Preliminary Analyses

In order to compare our results with past studies and the AASP normative means, we first looked at the amount of participants who scored in the extreme outer percentiles of the AASP normative distribution, which referred to scores that were below the 2nd percentile (AASP classification: “Much Less than Most People”) or above the 98th percentile (AASP classification: “Much More than Most People”) for each quadrant. Comparing percentages of extreme scores to those found in the AASP normative sample is common in most studies using the AASP (e.g., Rieke and Anderson 2009; Uljarević et al. 2014), and is a good complementary method in addition to statistically comparing group means.

In order to examine how gender and current presence of mental disorders might influence sensory scores independently from P-TD/SPX/MPX group effects, preliminary analyses consisting of *t* tests of independent samples were conducted to compare the AASP quadrant scores between genders and also between those with and without a current mental disorder. For these tests, all participants were pooled together and separated based only on the demographic variable in question.

### Primary Analyses

To statistically evaluate if sensory processing differed between SPX and MPX parents of ASD children and parents of TD children, we used a one-way multivariate analysis of covariance (MANCOVA) with diagnostic group (P-TD/SPX/MPX) as fixed factor, gender and presence of mental disorders as covariates, and the four AASP quadrant scores as dependent variables. Univariate analyses of covariance (ANCOVAs) were then performed for each quadrant, followed by post hoc analyses using Bonferroni’s correction method for multiple comparisons to determine group differences. *Atypical* s*ensory processing* was defined as significantly differing from the *“typical”* group (i.e., P-TD) in any quadrant score.

### Secondary Analyses

To determine if any modality in particular was responsible for the MANCOVA results, additional ANCOVAs were performed for each quadrant within each sensory modality (e.g., S. Seeking in the auditory modality, LR in the visual, etc.), followed by post hoc analyses using Bonferroni’s correction method for multiple comparisons when necessary. Participants were only included in this analysis if they had answered all questions in the specific modality and quadrant under investigation. Significance values were set at *p* ≤ .05 for all preliminary and main analyses, and all tests were performed using the software program, SPSS (Version 21.0).

## Results

### Descriptive Findings and Preliminary Analyses

**Fig. 1:**
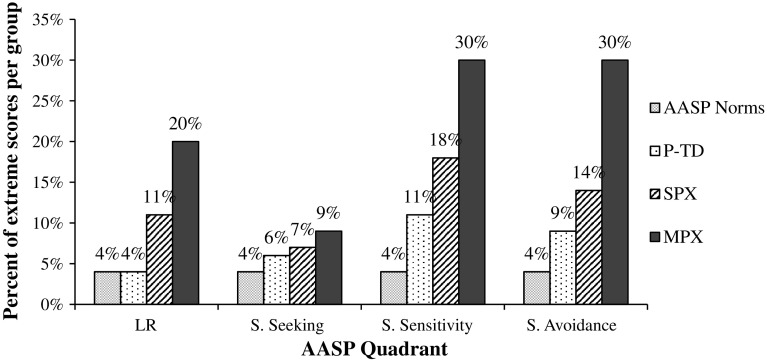
Percentage of each group that scored in the extreme outer percentiles of the AASP normative sample scores on four AASP quadrants. Information regarding the AASP norms and the cut-off scores defining the extreme outer percentiles for each quadrant are found in the AASP manual (Brown and Dunn 2002). *AASP* adolescent/adult sensory profile, *P-TD* parents of typically developing children, *SPX* parents of children with autism spectrum disorder (ASD) from simplex families, *MPX* parents of children with ASD from multiplex families

Figure 1 shows the percentage of each group that scored in the extreme outer percentiles in the four quadrants of the AASP. These percentages are compared to those found in the AASP normative sample (i.e., by definition, 4 % of the normative sample scored in the outer two percentiles in each quadrant). In all four quadrants, there was a visible linear trend across families (P-TD < SPX < MPX) in the amount of extreme scores.

After assessing the total amount of extreme sensory scores in each group, it was found that 53 % of MPX parents scored in the outer two percentiles in at least one AASP quadrant (28 % had extreme scores in only one quadrant, 15 % in two quadrants, 6 % in three quadrants, and 4 % in all four quadrants). In comparison, 27 % of SPX parents scored in the outer two percentiles in at least one quadrant (10 % in one quadrant, 12 % in two quadrants, and 5 % in three quadrants), and 20 % of P-TD parents scored in this range in at least one quadrant (14 % in one quadrant, 2 % in two quadrants, 4 % in three quadrants).

The preliminary *t* tests revealed that females scored significantly higher than males in two of the four AASP quadrants: S. Seeking, *t*(258) = 3.221, *p* = .001, and S. Sensitivity, *t*(258) = 3.382, *p* < .001; while S. Avoidance showed a trend: *t*(258) = 1.777, *p* = .07. In addition, parents currently diagnosed with a mental disorder scored significantly higher than parents without such diagnoses in three quadrants: LR, *t*(258) = 3.688, *p* < .0001; S. Sensitivity, *t*(258) = 4.185, *p* < .0001; and S. Avoidance *t*(258) = 4.129, *p* < .0001. Therefore, both gender and presence of a mental disorder were used as covariates in subsequent analyses.

### Primary Analyses

All dependent measures for the MANCOVA and the modality-specific ANCOVAs met assumptions for homogeneity (*p* > .05 for all Levene’s homogeneity of variance tests), thus permitting parametric analyses. Raw scores and standard deviations of each group are presented in Table 2.

**Table 2:** AASP quadrant scores among groups

Measure	P-TD^a^ *M* (*SD*)	SPX^b^ *M* (*SD*)	MPX^c^ *M* (*SD*)
Low registration	30.1 (7.1)	32.9 (8.1)	35.0 (9.6)
Sensation seeking	47.5 (7.4)	45.9 (7.7)	44.7 (7.7)
Sensory sensitivity	36.0 (9.3)	37.5 (10.5)	42.7 (10.5)
Sensation avoidance	36.4 (9.4)	38.4 (10.2)	43.1 (11.1)

*ASD* autism spectrum disorder, *AASP* adolescent/adult sensory profile, *P-TD* parents of typically developing children, *SPX* parents of children with ASD from simplex families, *MPX* parents of children with ASD from multiplex families

^a^ *n* = 85

^b^ *n* = 121

^c^ *n* = 54

**Fig. 2:**
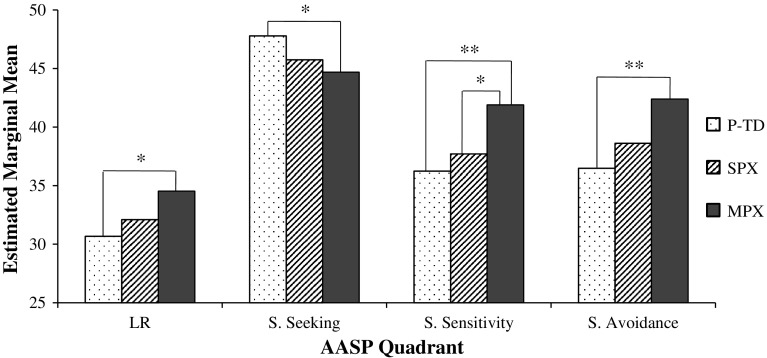
Pairwise comparisons of AASP quadrant scores among groups. Values shown are estimated marginal means with covariates evaluated at the following: presence of mental disorder = .3423 and gender = 1.14. *LR* low registration, *S* sensation/sensory, *AASP* adolescent/adult sensory profile, *P-TD* parents of typically developing children, *SPX* parents of children with autism spectrum disorder (ASD) from simplex families, *MPX* parents of children with ASD from multiplex families. *p < .05; **p < .01

The MANCOVA revealed significant differences in AASP scores among the P-TD, SPX, and MPX parent groups, *F*(8504) = 2.107, *p* = .034, Wilks’ Lambda = .936; *η*_*p*_^2^ = .32. Subsequent ANCOVAs found that scores significantly differed among parent groups in all four sensory quadrants: LR, *F*(2255) = 3.796, *p* = .024, *η*_*p*_^2^ = .029; S. Seeking, *F*(2255) = 3.246, *p* = .041, *η*_*p*_^2^ = .025; S. Sensitivity, *F*(2255) = 5.649, *p* = .004, *η*_*p*_^2^ = .042; and S. Avoidance, *F*(2255) = 5.825, *p* = .003, *η*_*p*_^2^ = .044. Figure 2 illustrates group differences after correcting for multiple comparisons using Bonferroni’s method.

### Secondary Analyses

As shown in Table 3, results of the modality-specific ANCOVAs and Bonferroni corrected post hoc analyses revealed that MPX parents scored significantly higher than both SPX and P-TD parents in the auditory total score, auditory S. Sensitivity, auditory S. Avoidance, visual total score, visual LR, and visual S. Avoidance (all *p*s < .005), and MPX parents also scored higher than P-TD parents in auditory LR, activity LR, touch S. Sensitivity, and visual S. Sensitivity (all *p*s < .05). In addition, it was found that SPX parents scored higher than P-TD in auditory LR (*p* < .01), while P-TD parents scored higher than SPX in the movement total score (*p* < .05).

**Table 3:** AASP quadrant scores separated by sensory modality

Modality	P-TD	SPX	MPX	*F*	*p*	Post hoc comparisons	*η*_*p*_^2^
	Means^a^						
Auditory total	26.20	27.80	31.46	7.187	**.001**	**MPX** **>** SPX*, P-TD***	**.053**
LR	6.40	7.28	7.63	4.831	**.009**	MPX > P-TD*; SPX > P-TD*	**.037**
S. Seeking	5.65	5.44	5.19	1.157	.316	n.s.	n.s.
S. Sensitivity	7.51	7.89	9.43	7.476	**.001**	**MPX** **>** SPX***, P-TD***	**.056**
S. Avoidance	6.70	7.37	8.91	9.100	**.0001**	**MPX** **>** SPX**, P-TD***	**.067**
Activity total	26.68	27.89	28.41	2.263	.106	n.s.	n.s.
LR	6.28	7.04	7.47	3.808	.023	MPX > P-TD*	.029
S. Seeking	9.23	8.92	8.39	2.729	.067	n.s.	n.s.
S. Sensitivity	2.99	3.30	3.20	1.611	.202	n.s.	n.s.
S. Avoidance	8.22	8.75	9.05	2.126	.121	n.s.	n.s.
Movement total	21.02	19.56	20.81	3.345	.037	P-TD > SPX*	.026
LR	4.36	4.26	4.76	1.330	.266	n.s.	n.s.
S. Seeking	7.49	6.86	6.76	3.411	.035	n.s.	n.s.
S. Sensitivity	7.72	6.98	7.47	2.409	.092	n.s.	n.s.
S. Avoidance	1.62	1.45	1.53	.893	.411	n.s.	n.s.
Taste/smell total	20.74	20.40	21.41	.932	.395	n.s.	n.s.
LR	4.03	3.84	4.06	.493	.611	n.s.	n.s.
S. Seeking	9.41	8.54	9.15	3.116	.046	n.s.	.024
S. Sensitivity	2.12	2.47	2.60	2.357	.097	n.s.	n.s.
S. Avoidance	5.40	5.57	5.57	.252	.778	n.s.	n.s.
Touch total	31.29	32.08	34.39	2.138	.120	n.s.	n.s.
LR	6.01	5.91	6.09	.155	.857	n.s.	n.s.
S. Seeking	9.62	9.83	9.22	1.080	.341	n.s.	n.s.
S. Sensitivity	8.62	8.83	10.14	3.783	.024	MPX > P-TD*	.029
S. Avoidance	7.24	7.68	8.44	2.681	.070	n.s.	n.s.
Visual total	24.62	26.02	28.94	8.434	**.0003**	**MPX** **>** SPX*, P-TD***	**.062**
LR	3.67	3.77	4.56	6.702	**.001**	**MPX** **>** SPX***, P-TD***	**.050**
S. Seeking	6.44	6.24	6.09	.793	.454	n.s.	n.s.
S. Sensitivity	7.33	8.27	9.07	6.493	**.002**	**MPX** **>** P-TD***	**.048**
S. Avoidance	7.33	7.81	8.90	5.913	**.003**	**MPX** **>** SPX*, P-TD***	**.044**

Bold font indicates strongly significant (*p* < .01) differences in modalities between groups

*AASP* adolescent/adult sensory profile, *LR* low registration, *S.* sensation/sensory, *P-TD* parents of typically developing children, *SPX* parents of children with ASD from simplex families, *MPX* parents of children with ASD from multiplex families, *n.s.* non-significant

^a^Estimated marginal means with covariates evaluated at the following: presence of mental disorder = .3424 and gender = 1.14

* *p* < .05; ** *p* < .01; *** *p* ≤ .005

## Discussion

This study investigated the hypothesis that sensory processing, as measured by the adolescent/adult sensory profile, would differ among parents of typically developing children and parents of children with ASD from SPX and MPX families. Results supported this hypothesis by showing that, after controlling for gender and mental disorders, MPX parents scored significantly lower than P-TD parents in Sensory Seeking, and significantly higher than P-TD parents in the Low Registration, Sensation Avoidance, and Sensory Sensitivity quadrants of the AASP. Upon investigating each modality separately, it was found that the primary results were influenced mostly by scores in the auditory and visual modalities. Differences between SPX and MPX parents reached significance in the Sensory Sensitivity quadrant of the primary analyses, and also in various auditory and visual quadrant scores of the secondary analyses. Our findings that parents with high genetic liability for ASD-related genes (MPX) had more sensory atypicalities than parents with low (SPX) or no (P-TD) genetic liability for ASD suggest that atypical sensory processing may contribute to the genetic susceptibility for ASD. Our conclusions align with recent genetics research suggesting that sensory processing atypicalities may share genetic influences with ASD (DeLorey et al. 2011; Peñagarikano et al. 2011; Tavassoli et al. 2012). Whether sensory processing atypicalities represent an increased risk for ASD specifically, or instead an increased risk for a range of mental disorders (only one of which is ASD), is yet to be verified.

Results from the present study confirm Uljarević et al.’s (2014) past findings that parents of children with ASD had more sensory processing atypicalities than the norm. Although we replicated these general findings, effects in our study were found only in MPX parents of ASD children, while SPX parents scored similarly to P-TD parents. Uljarević et al.’s finding that 44 % of mothers of ASD children scored in the extreme outer percentiles (i.e., > 2 SDs outside the norm) in at least one quadrant is somewhat similar to our results concerning the percentage of MPX parents that scored in the extreme outer percentiles (53 %), but in contrast to results from the SPX (27 %) and P-TD (20 %) parent groups in our study. It is crucial to note that these percentages are not corrected for potentially confounding variables, namely gender and mental disorders, and therefore it is difficult to make valid comparisons based on these data alone. However, after stringent statistical testing including controlling for these variables, we continued to find effects in MPX parents only, whereas scores from SPX parents did not statistically differ from P-TD parents in any primary analysis. Our results showing increased sensory processing atypicalities in MPX parents compared to SPX and P-TD parents offer genetic explanations for Uljarević et al.’s findings, and also support De la Marche et al.’s (2012) conclusion that decreased Sensation Seeking may be an intermediate phenotype of ASD.

Our findings that the auditory and visual modalities showed the largest group effects may be due the crucial involvement of auditory and visual processing in social communication. Successful interpretation of social communication relies on sufficient processing of auditory cues, visual cues, and audio–visual integration, thus emphasizing the relevance of atypical auditory and visual processing in ASD studies. In addition to ASD-specific explanations, it is also possible that group effects were found mostly in the auditory and visual modalities due to factors relating to self-report sensory questionnaires. Considering that reactions experienced in the auditory and visual modalities are verbalized more often than other modalities, participants may have more difficulty in identifying, recalling, or reporting reactions experienced in the other senses.

Our preliminary findings concerning effects of mental disorders are consistent with previous studies that found a relationship between sensory processing abnormalities and various types of mental disorders and disorder symptoms (Ashburner et al. 2008; Brown et al. 2002; Engel-Yeger and Dunn 2011; Engel-Yeger et al. 2013; Mangeot et al. 2001; Rieke and Anderson 2009). Given that (a) ASD probands and their families have an increased chance of having other heritable mental disorders in addition to ASD (e.g., Bolton et al. 1998; Daniels et al. 2008; DeLong and Nohna 1994; Piven and Palmer 1999) and that (b) many of these mental disorders are also related to atypical sensory processing (e.g., Brown et al. 2002; Engel-Yeger et al. 2013; Rieke and Anderson 2009), it remains uncertain whether the increased sensory processing atypicalities observed in MPX parents in our study are related to an increase in genetic susceptibility for ASD specifically, or instead are related to an increased risk for a range of mental disorders. Regardless of whether sensory processing atypicalities are *specific* to ASD, our findings nevertheless support previous evidence implying that abnormal sensory processing and ASD may share genetic influences.

Preliminary results from this study support recent studies showing that females scored significantly higher than males on sensory processing questionnaires (Engel-Yeger 2012; Horder et al. 2014; Tavassoli et al. 2014), which contributes to the accumulating evidence demonstrating gender differences in sensory processing (Velle 1987). In addition to gender differences that are specific to the female sensory systems, it is also possible that our results concerning gender effects may be partially due to cognitive differences or differences in self-disclosure. For instance, females are found to have a better memory for recognizing and identifying odors (Brand and Millot 2001) and are more likely to disclose personal information than males (Dindia and Allen 1992). Although these explanations might account for the gender effects found in our study and are interesting for future investigations, they cannot explain our main conclusions concerning differences in ASD genetic liability, considering that gender was distributed evenly among all three groups and was controlled for in all main analyses.

Throughout all analyses, there was a linear (though at times non-significant) trend in which atypical sensory scores increased as the amount of presumed genetic liability for ASD increased: P-TD parents had fewer sensory atypicalities than SPX parents, who in turn had less sensory atypicalities than MPX parents. A likely explanation for the slight increase in scores in the SPX group is that some parents who were originally designated to this group might in fact have ASD genetic mechanisms more similar to the traditional assumptions of MPX families (i.e., familial ASD-related traits) than those of SPX (i.e., isolated genetic mutations), which is a common concern in SPX/MPX studies (e.g., Klei et al. 2012). In addition to general uncertainty of mental disorder status of all biological relatives, another factor contributing to group misplacement could be the “stoppage effect,” which occurs when a family ceases child-bearing after an ASD diagnosis is discovered in one of their children (Jones and Szatmari 1988). This effect questions a family’s SPX classification, as it is unknown how future children would have developed. Another possible explanation for group misplacement is that some relatives within SPX families could have pronounced ASD traits without an official ASD diagnosis, and therefore would not be recognized in our family history questionnaire. SPX families with prevalent ASD traits (albeit no official diagnoses) would better fit the genetic profile of MPX families than that of SPX. Considering these various situations, it is possible that some SPX parents may have had unrealized MPX status, thus causing a slight increase in overall sensory atypicalities in this group.

Past studies investigating intermediate phenotypes in relatives of ASD individuals have already identified numerous heritable traits that may increase genetic vulnerability for ASD, including social/emotional abnormalities (e.g., Gerdts et al. 2013), pragmatic language impairments (e.g., Whitehouse et al. 2007), stereotyped behaviors (e.g., Piven et al. 1997), and even certain personality traits (Losh et al. 2008; Murphy et al. 2000; Piven et al. 1994). Findings from the present study suggest that atypical sensory processing might be an additional heritable trait contributing to ASD susceptibility. Assuming that each heritable trait of ASD is expressed as a result of variations in specific genes related to ASD susceptibility (Piven 2001), then it is plausible that an increase in the number of inherited ASD-related genetic variations would additively increase vulnerability of developing ASD. Along these lines, Klei et al. (2012) found that many common genetic variations that have small effect in isolation, have large effects on ASD susceptibility when acting additively. These additive effects were found more often in relatives from MPX ASD families (60 %) than from SPX ASD families (40 %), thus implying that ASD cases from MPX families may result largely from the additive effect of numerous ASD-related genetic variations of small influence. It may be that some of these common genetic variations give rise to the ASD-like traits often observed in relatives of ASD probands. Considering that sensory processing atypicalities are now part of the ASD diagnostic category of restricted repetitive behaviors (APA 2013) and are strongly associated with autistic traits in both ASD (Boyd et al. 2009; Wigham et al. 2015) and non-ASD populations (Robertson and Simmons 2013; Horder et al. 2014), the results from the present study imply that atypical sensory processing might be an additional potential BAP trait worthy of future investigations, which could further aid in the search for genetic variations responsible for ASD.

In addition to theoretical implications concerning atypical sensory processing and ASD genetic liability, the current study also has important practical implications. Regardless of the exact causes behind these results, our findings show that many parents of ASD children perceive the sensory world differently than most people, which, in extreme cases, could result in difficulties in many areas of daily functioning. According to personal accounts from individuals with ASD, severe sensory reactions can encourage social withdrawal and reduce participation in many activities (Grandin 1992; Kirby et al. 2015), a consequence which influences the individual’s general social/communication functioning. These consequences may also apply to some MPX parents, considering that the overall pattern of sensory atypicalities seen in MPX parents in this study (i.e., lower than controls in S. Seeking and higher in the remaining three quadrants) directly corresponds to the sensory pattern found in adults diagnosed with ASD (Crane et al. 2009).

Apart from affecting social functioning, sensory processing difficulties have also been associated with anxiety symptoms (Engel-Yeger and Dunn 2011; Kinnealey and Fuiek 1999), depression symptoms (Liss et al. 2005; Kinnealey and Fuiek 1999), sleep quality (Engel-Yeger and Shochat 2012), and even physical health symptoms (Benham 2006). Although these associations do not imply causation, they nevertheless demonstrate that many parents of ASD children who have sensory symptoms likely have additional problems affecting their well-being. While it is standard practice to treat sensory symptoms in children diagnosed with ASD, the present study suggests that their parents could also benefit from understanding and alleviating their own sensory difficulties. This would not only improve the parent’s well-being, but could also improve their child’s well-being, by enabling a more nurturing and enriching environment in which the child could develop into his or her highest potential.

Although this study’s sample size was relatively large [i.e., three times the size of past studies using the AASP in ASD relatives (De la Marche et al. 2012; Uljarević et al. 2014)], thus emphasizing the validity of these findings, there are a few limitations concerning the sample that should be addressed. First, we relied on self-reports of mental diagnoses of the participant, their children, and—due to our expanded definition of MPX families—their biological relatives. Validity of these diagnoses is crucial, as the diagnostic statuses of the participant’s child and biological relatives created the distinction among the three study groups, and the participant’s own diagnoses were found to have large effects on AASP scores in preliminary analyses. Diagnostic evaluations by clinicians of the parents, their children, and their relatives would have been better to ensure accuracy of ASD diagnoses in the ASD groups, and also to guarantee a well-controlled comparison group. However, we did make an effort to alleviate the latter concern by excluding parents from the P-TD group who had suspicions that their child might have ASD or who reported that they had a biological relative with ASD. In addition, it is important to note that participants in the ASD-affected parent groups were recruited from official ASD organizations where diagnoses by a licensed professional are administered, required, or assumed. Therefore, although ASD diagnoses of the children with ASD could not be directly confirmed, our manner of recruitment increases confidence that a participant’s child likely received a confirmed diagnosis of ASD by a licensed professional if the parent reported so on the questionnaire.

Clinical diagnostic evaluations of the participants would have not only confirmed their current mental disorders, but could have also provided information regarding their sub-clinical disorder *symptoms*. As sensory processing abnormalities were found to be associated with symptoms of several mental disorders (e.g., Dar et al. 2012; Engel-Yeger and Dunn 2011; Horder et al. 2014; Liss et al. 2005), it remains uncertain whether subclinical symptoms in the parents could have partially explained our results. Addressing this limitation in future research is imperative in order to better understand the extent to which sensory processing abnormalities are specific to ASD genetic liability.

A general limitation of using sensory questionnaires is the reliance on self-reported subjective reactions to sensory experiences, which requires honest and reliable introspection. Furthermore, the AASP has been criticized for including sensory items that are associated with affective or social reactions (Tavassoli et al. 2014). For these reasons, supplementary sensory processing measurements involving objective physiological tests are recommended to accompany self-report questionnaires, although this was unfortunately not an option for the present online study. Despite potential limitations of the AASP, the present study nonetheless produced significant findings, which is a valuable advancement towards understanding how atypical sensory processing may relate to the genetic liability for ASD.

Future research should replicate the present study’s results with use of professional diagnostic evaluations and additional objective sensory measurements, such as measuring behavioral or physiological reactions to sensory stimuli. Given that this study used online questionnaires to understand traits contributing to ASD genetic liability, evident next steps also include genetic association studies to discover the genes underpinning our results, similar to the pilot study by Tavassoli et al. (2012).

Through investigating the many traits of the broader autism phenotype and eventually discovering their genetic etiology, we can improve genetics research and gradually move closer toward a global understanding of ASD. Findings from the present study contribute to this effort by suggesting that atypical sensory processing may be one of the heritable traits contributing to ASD susceptibility, which is worthy of future investigations.

## Footnotes

1 In this context, a mental disorder refers to any psychological or behavioral syndrome recognized in the DSM (APA 2013) or *International Statistical Classification of Diseases and Related Health Problems* (World Health Organization 1992) that causes “clinically significant disturbance in an individual’s cognition, emotion regulation, or behavior” (APA 2013).
